# Improving muscle recruitment via multi-electrode transcutaneous spinal cord stimulation using automated selectivity-driven algorithms

**DOI:** 10.1063/5.0298057

**Published:** 2026-03-09

**Authors:** Mouhamed Zorkot, Solaiman Shokur, Riccardo Carpineto, Silvestro Micera, Mohamed Bouri

**Affiliations:** 1 Translational Neural Engineering (TNE) Lab, Neuro-X Institute, EPFL, Geneva, Switzerland; 2 Rehabilitation and Assistive Robotics (REHAssist) Group, EPFL, Lausanne, Switzerland; 3 Bioelectronics and Bioengineering Area, The BioRobotics Institute and Department of Excellence in Robotics and AI, Scuola Superiore Sant'Anna, Pisa, Italy; 4 Department of Clinical Neurosciences, University Hospital of Lausanne and University of Lausanne, Lausanne, Switzerland; 5 Modular Implantable Neurotechnologies (MINE) Laboratory, Università Vita-Salute San Raffaele and Scuola Superiore Sant'Anna, Milan, Italy

## Abstract

Spinal cord injury (SCI) severely impairs motor function and quality of life. Transcutaneous spinal cord stimulation (tSCS) has emerged as a promising non-invasive neuromodulation technique to restore voluntary motor function by engaging spinal circuits below the lesion. While standard tonic tSCS with a single electrode at T11–T12 offers limited gait-specific selectivity, recent studies show that multi-electrode configurations can recruit better proximal and distal muscles on the ipsilateral side. However, clinical translation of such approaches is still limited due to individual variability and the need for time-consuming calibration procedures that rely on manual electrode placement and offline analysis. We aim to enhance the selectivity of tSCS in multi-electrode configurations and to implement online spinal reflex detection and automated algorithms for personalizing stimulation parameters, enabling selective activation of target muscle groups. We propose an automated protocol with online spinal reflex detection and muscle response analysis and developed two algorithms based on near-instantaneously generated online data to determine the optimal electrode position and stimulation amplitude to maximize the selective recruitment of target muscle groups. The approach was tested in **14 healthy participants** in the supine position using two distinct multi-electrode configurations: **midline configuration** employs three electrodes aligned rostrocaudally along the spinal midline to target proximal, distal, and all lower limb muscle groups and **bilateral configuration** employs six electrodes, with three electrodes positioned rostrocaudally and symmetrically on each side of the spinal midline to target six muscle groups (rostrocaudal and ipsilateral). Both setups integrated an automated posterior root-muscle reflex protocol with online spinal reflex detection. Electromyography (EMG) data recorded during stimulation were processed by two independent algorithms: (1) the **ranking-based approach (RBA)**, which applies rule-based hierarchical criteria to rank electrodes based on spinal reflex responses, and (2) **selectivity-driven approach (SDA)**, which computes a selectivity index to quantitatively assess muscle activity. For each target muscle group, the output of the algorithms is the selection of the optimal electrode and stimulation amplitude that achieves the most selective recruitment. We found that both developed approaches contribute to enhancing the rostrocaudal and ipsilateral selectivity in multi-electrode tSCS. We suggest that SDA is more suitable for selectively recruiting target muscle groups, as it quantifies selectivity based on graded EMG responses, while the RBA is well-suited for rapid, generalized applications, such as the conventional single-electrode tSCS to maximize overall muscle activation. Furthermore, our results challenge common assumptions about tSCS selectivity, including rostrocaudal recruitment of proximal/distal muscles and ipsilateral activation. Indeed, in the midline configuration, 9/13 participants showed greater recruitment with the T11–T12 electrode; when in the bilateral configuration, 5/11 had stronger contralateral leg activation in at least one of the electrodes, possibly due to anatomical variability of the spinal cord. These deviations highlight the value of online spinal reflex detection and automated algorithms for optimizing single- and multi-electrode tSCS, reducing reliance on manual electrode placement and accounting for inter-subject variability, thereby enabling more targeted neuromodulation and personalized gait rehabilitation for SCI and other neurological conditions.

## I. INTRODUCTION

Spinal cord injuries (SCI) remain a major global health challenge, with an estimated incidence of approximately 23.8 cases per × 10^6^ individuals and a continuously increasing number of years lived with disability.^1,2^ These injuries often result in severe motor impairment and a lifelong dependence on intensive rehabilitation interventions.^2^ Although current therapeutic approaches, ranging from conventional physical rehabilitation to advanced technologies such as robotic exoskeletons and functional electrical stimulation (FES), can support partial motor improvements,^3^ they still present significant limitations in restoring natural and independent movement. Reported constraints include reduced physiological gait patterns, increased muscle fatigue, passive or involuntary movements, and no significant functional improvement.^3–5^ Given the crucial role of the spinal cord in the central nervous system and motor control, spinal cord stimulation (SCS) has emerged as a promising approach to gait rehabilitation.^6,7^

SCS encompasses both invasive methods, with epidural spinal cord stimulation (eSCS), and non-invasive approaches, with transcutaneous spinal cord stimulation (tSCS). Both techniques modulate the excitability of spinal neural circuits by activating proprioceptive afferent fibers in the dorsal roots, thus facilitating voluntary lower limb movement.^6–9^ During recent decades, eSCS has been increasingly investigated in human clinical trials, with the primary objective of enabling the recovery of voluntary locomotor control after SCI,^6–9^ facilitating recovery through the reorganization of residual neural pathways.^10^

Tailored activity-based protocols and precise electrode placement have enabled individuals with sensorimotor deficits to regain more natural leg control, supporting functional activities such as standing, walking, and stair climbing.^6,7^ Moreover, optimization algorithms have been explored in both animal models^11,12^ and humans^13^ to facilitate clinical translation, personalize stimulation strategies, and refine muscle recruitment control to maximize therapeutic efficacy.^11–13^ These advancements represent a breakthrough in neurorehabilitation; however, the clinical application of eSCS is limited by its invasive nature, which requires surgical intervention.

In contrast, tSCS is emerging as a more accessible alternative, offering a non-invasive intervention utilizing mechanisms similar to those of eSCS.^14^ tSCS targets the same neural subpopulations in the spinal cord by activating large and medium-diameter afferent fibers and, when delivered at sub-motor-threshold intensities, modulates the excitability of the spinal network without directly eliciting action potentials.^15^ The stimulation propagates through the dorsal root afferents,^15^ and when combined with activity-based therapy, tSCS has demonstrated functional improvements in individuals with SCI.^16–19^ Although supported by a smaller evidence base than eSCS, tSCS has been reported to produce qualitatively comparable improvements.^17^

Recent research with tSCS also focuses more on SCI and highlights significant benefits of tSCS, including improved locomotor activity,^20^ limb mobility,^19^ and restoration of walking function.^16,17^ Standard tSCS clinical protocols typically involve placing a surface electrode manually on the T11–T12 vertebral level, and the stimulation amplitude is calibrated to 10% below the motor threshold, as determined by the posterior root muscle reflex test (PRM).^18,19^ The PRM test plays a pivotal role in assessing the excitability of the spinal cord during tSCS. By activating proprioceptive fibers in the dorsal roots, the test elicits short-latency spinal reflexes, leading to motor neuron activation and detectable muscle responses via electromyography (EMG).^18^

When compared with eSCS, computational modeling indicates that only 8 % of the current delivered during tSCS reaches the cerebrospinal fluid (CSF), owing to the multiple intervening tissue interfaces, whereas approximately 80%–90% of the current enters the CSF during eSCS. These pronounced differences in current distribution and electrode proximity to neural structures result in distinct levels of spinal circuit selectivity and necessitate different stimulation intensities across the two modalities.^14^ Nevertheless, although tSCS generally provides less focal activation than eSCS,^14^ the magnitude and consistency of its selective muscle recruitment remain insufficiently established. At the lumbar level, most studies involving tSCS in individuals with SCI have employed single-electrode stimulation. However, building on the rostrocaudal anatomical organization of motor neuron pools that innervate leg muscles,^6,21,22^ recent studies have demonstrated the potential of tSCS to achieve selective recruitment of proximal and distal muscles along the rostrocaudal axis,^23,24^ as well as ipsilateral selectivity for targeting unilateral limbs,^25^ by stimulating specific sensorimotor networks.

Recently, Bryson *et al.*^26^ explored a multi-electrode tSCS approach using six 3.2 cm electrodes arranged rostrocaudally and unilaterally, laterally to the midline at T11/T12 vertebrae. Their study showed that single-electrode tSCS often lacks specificity, activating both proximal and distal leg muscles simultaneously. In contrast, placing a cathode at T10/T11 (targeting L1–L3) selectively activated proximal muscles, while positioning it at T12/L1 (targeting L4–S3) preferentially recruited distal muscles. These findings reinforce the potential for selective muscle recruitment via optimized electrode placement and stimulation intensity.

Optimizing electrode placement for tSCS is challenging and time-consuming, often leading to suboptimal results due to individual variability across subjects and sessions.^27^ Traditional methods rely on manual electrode positioning and offline muscle activity analysis, limiting the precision and efficacy of selective activation.^27^ To address this, Salchow-Hommen *et al.*^27^ introduced a binary algorithm designed for automated calibration in a multi-electrode spinal configuration. This approach aims to identify the electrode position and stimulation amplitude that activate the largest number of lower-limb muscles. However, while effective in maximizing muscle activation, this method does not prioritize enhancing tSCS selectivity.

In this context, the present work directly addresses a critical limitation in the field: the low selectivity of conventional single-electrode tSCS and the difficulty of achieving consistent muscle targeting with multi-electrode configurations. These challenges arise from intra- and inter-individual anatomical variability, dependence on manual electrode placement, and reliance on offline analysis.^27^ This study aims to enhance the selectivity of multi-site tSCS by implementing online spinal reflex detection and automated algorithms that personalize stimulation parameters, enabling selective activation of target muscle groups.

**FIG. 1.**
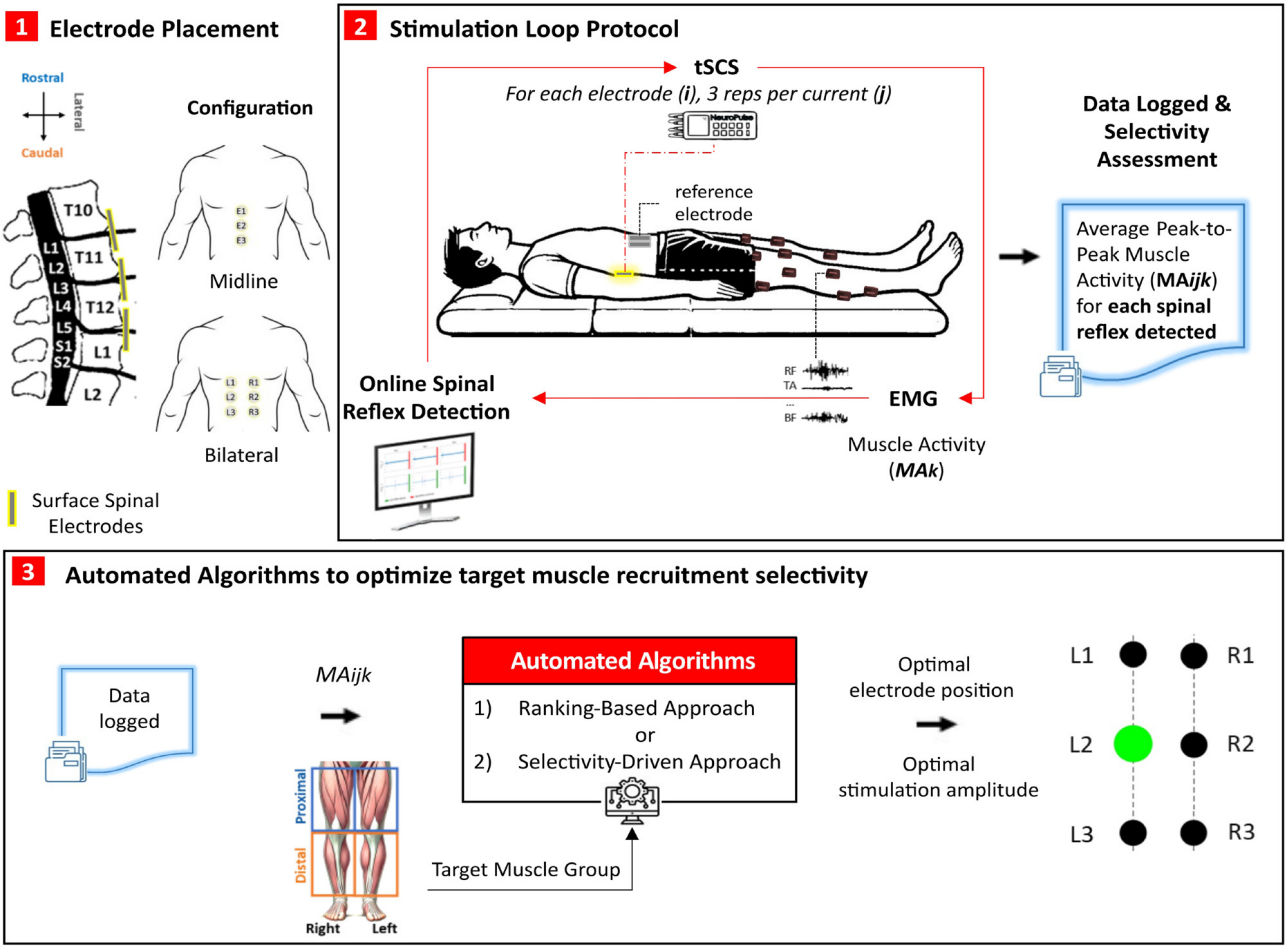
Framework of the online spinal reflex and automated algorithms for enhancing muscle group selectivity in multi-electrode tSCS. (1) Electrode placement: Electrodes (*i*) were positioned over the T10–T11, T11–T12, and T12–L1 vertebral levels in two configurations: (i) *Midline*—three electrodes (E1, E2, E3) aligned rostrocaudally over the spinal midline; (ii) *Bilateral*—six electrodes placed rostrocaudally and symmetrically on both sides of the midline (L1–L3 on the left, R1–R3 on the right). (2) Stimulation loop protocol: For each configuration, tSCS was applied in the supine position using the PRM reflex test. Stimulation amplitudes ranged from 10 mA below the visually identified reflex threshold to the maximum tolerated intensity. Each electrode delivered biphasic double-pulse stimulation every 5 s, with three repetitions per amplitude ( $I_{j}$). Bilateral EMG signals were recorded from proximal and distal leg muscles to extract muscle activity ( ${\text{MA}}_{k}$). For each electrode, amplitude, and muscle, the presence or absence of spinal reflexes was detected online and the average peak-to-peak muscle activity observed in at least two out of three repetitions were averaged and stored ( ${\text{MA}}_{\text{ijk}}$). (3) Automated algorithms to optimize selective recruitment of target muscle groups: Immediately following the online protocol, two automated algorithms were applied to optimize target muscle group recruitment using the near-instantaneously generated data, independently for each target muscle group. (a) The ranking-based approach (RBA) applies a hierarchical set of prioritized rules to rank electrodes based on reflex recruitment, whereas (b) the selectivity-driven approach (SDA) evaluates electrode performance using a quantitative selectivity index (SI). The output of both algorithms is the selection of the most selective electrode position ( $\varepsilon_{p}$) and stimulation amplitude (*Istim*) for maximizing target muscle group recruitment.

**FIG. 2.**
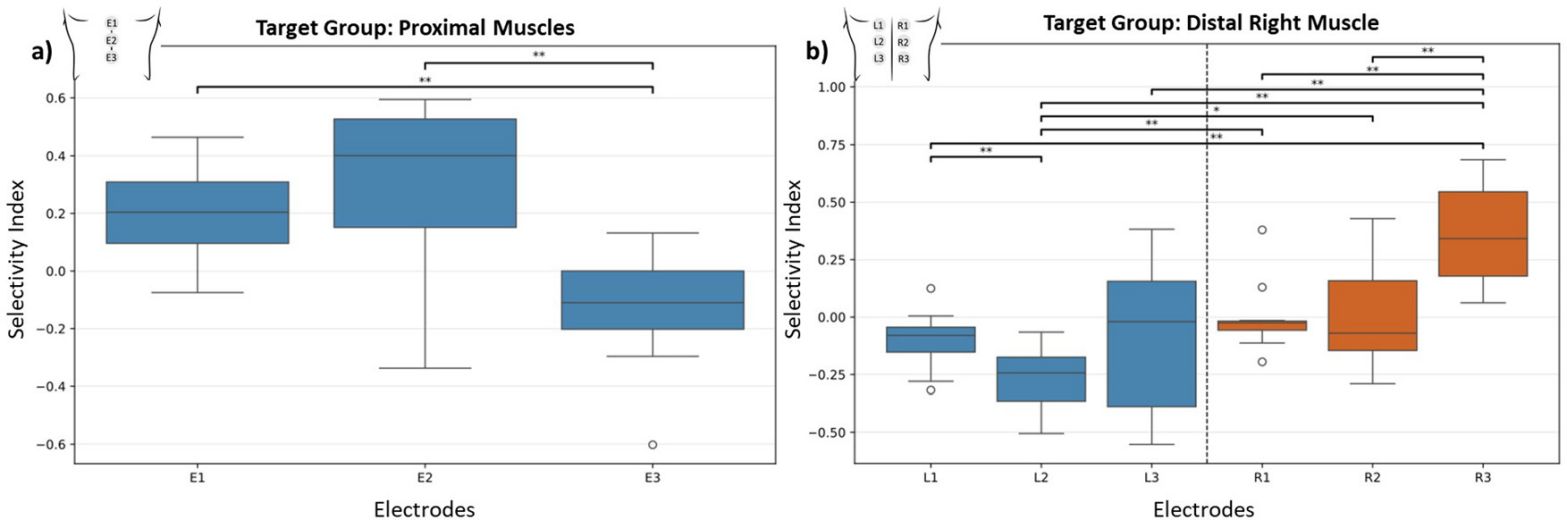
Selectivity index for target muscle groups: (a) Midline configuration targeting the proximal muscle group ( S=13) and (b) bilateral configuration targeting the right distal muscle group ( S=11). Error bars represent the standard deviation across participants.

**FIG. 3.**
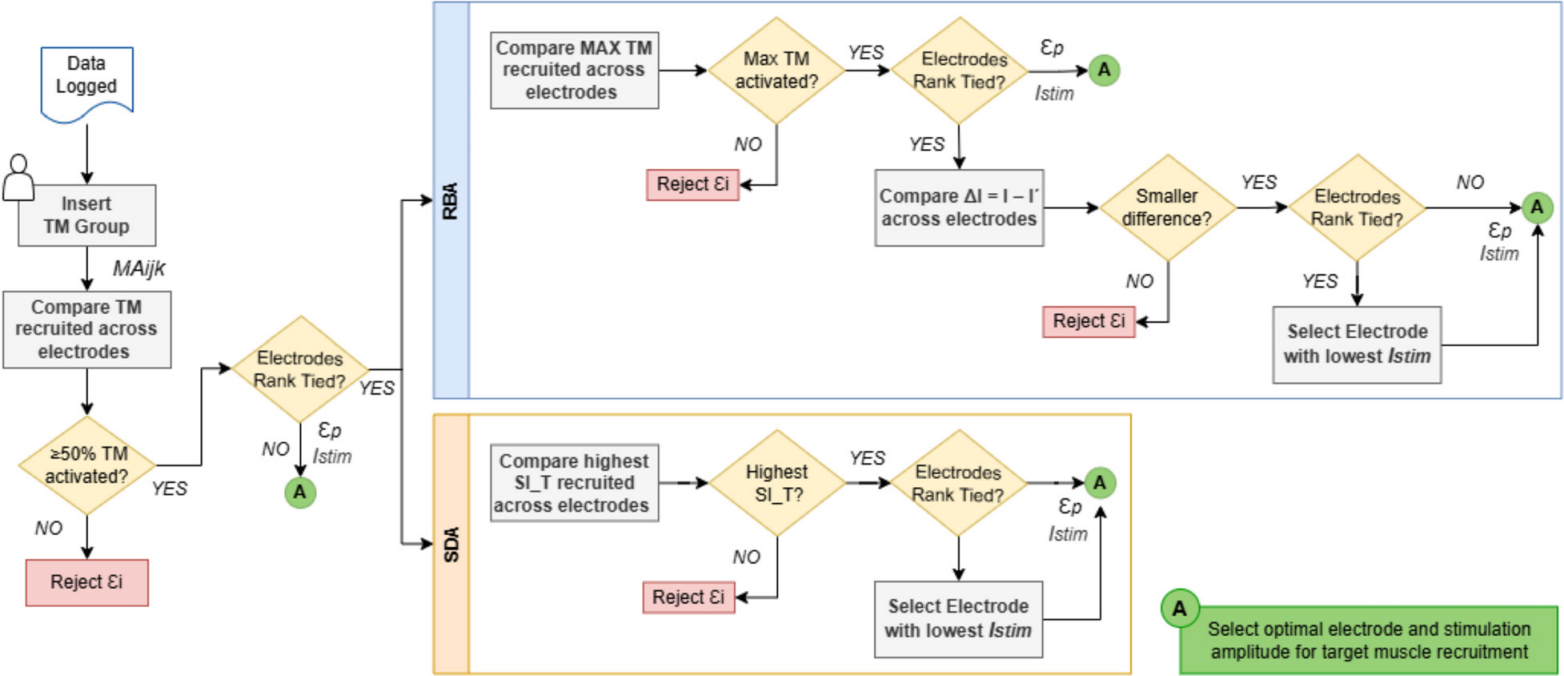
Flowchart of the two automated algorithms developed to optimize multi-electrode tSCS selectivity for targeted muscle (TM) group recruitment. Both algorithms determine the optimal electrode position ( $\varepsilon_{p}$) and stimulation amplitude (*Istim*) for selective activation of the target muscle groups. The logged data, used as input to the automated algorithms, consist of the presence or absence of spinal reflexes and the average peak-to-peak EMG responses for each electrode ( $\varepsilon_{i}$) and stimulation amplitude, computed for each muscle ( ${\text{MA}}_{\text{ijk}}$). The **ranking-based approach (RBA)** applies a rule-based hierarchy prioritizing reflex detection and threshold efficiency, while the **selectivity-driven approach (SDA)** computes a selectivity index ( $SI_{T}$) to quantify muscle-specific recruitment.

To achieve this goal, we developed and validated an automated stimulation protocol that integrates online spinal reflex detection and automated algorithms, enabling individualized recruitment of proximal and distal muscle groups in either leg using multi-electrode tSCS (Fig. 1). Participants underwent the PRM reflex test in the supine position using both midline and bilateral electrode configurations (Fig. 1-1). During stimulation, lower limb muscle activity was continuously monitored via EMG to detect spinal reflexes online and to quantify muscle responses across electrode configurations and stimulation amplitudes (Fig. 1-2). Immediately following completion of the online protocol, two automated algorithms were applied to optimize target muscle group recruitment using the near-instantaneously generated data: (1) a binary ranking-based approach **(RBA)**, adapted from Salchow-Hömen *et al.*^27^ and optimized for selectivity; and (2) a novel selectivity-driven approach **(SDA)**, which quantitatively evaluates EMG responses to maximize selective activation. These independent algorithms determine the optimal electrode configuration and stimulation amplitude required for selective recruitment of each target muscle group (Fig. 1-3).

The hypotheses underlying this framework are as follows: (i) automatic and online evaluation of spinal reflexes will facilitate tSCS calibration by enabling near-instantaneous assessment of muscle recruitment and reducing inter-subject variability; (ii) automated algorithms will identify the optimal spinal electrode position and stimulation current required for selective recruitment of the targeted muscle groups; and (iii) the automated framework will offer greater reliability than manual procedures and reduce calibration time.

Overall, these advances aim to strengthen the clinical feasibility of tSCS and to support the development of more efficient, selective, and personalized neuromodulation strategies for motor rehabilitation.

## II. RESULTS

### A. Online spinal reflex detection and automated multi-electrode tSCS framework

#### 1. Validation of online spinal reflex detection

To evaluate the accuracy of the online PRM detection method, online classifications were compared against offline ground-truth analyses across 100 acquired reflex events. The initial implementation of the online detector achieved an accuracy of 87%, with the remaining misclassifications primarily linked to low-amplitude stimulation artifacts and the absence of precise timing synchronization between stimulation delivery and EMG acquisition.

Although several thresholds were evaluated (50%, 60%, 70%, 80%, and 90%), the value of 70% delivered the best detection performance without any need for additional tuning. This value was validated offline across participants to provide a conservative and robust criterion for reflex detection. The consistency between online and offline performance confirms that the fixed 70% suppression threshold generalizes reliably across muscles and participants, making it suitable for automated detection.

#### 2. Time requirements of the automated multi-electrode tSCS protocol

Based on our experiments, in which two experienced researchers performed the palpation-based electrodes placement, manual identification of a single electrode position typically required approximately 10 min to evoke consistent responses across all lower-limb muscles. When extended to multi-electrode configurations, an approach that remains relatively recent in the tSCS literature, no published studies report detailed timing metrics. However, drawing from our laboratory experience, manual calibration for a midline configuration using three electrodes generally required 15–20 min, while bilateral multi-site configuration required 20–25 min. These intervals include palpation of anatomical landmarks, electrode placement, adjustments due to inconsistent reflexes, and repeated PRM testing until a stable response is achieved. Previous observations in the literature support this variability: Bryson *et al.*^26^ reported that electrode repositioning was necessary in multiple participants, highlighting the inherent difficulty in achieving reliable targeting using palpation alone. Moreover, Salchow-Hömen *et al.*^27^ noted that manual multi-electrode placement often requires repeated adjustments of several centimeters, reflecting a highly operator-dependent process.

The proposed automated calibration protocol standardizes stimulation delivery and reduces the need for iterative manual repositioning. With a pause of 5 s per double pulse for each electrode, the automated midline configuration (15 current of stimulation × 3 repetitions) required 11.25 min, whereas the bilateral configuration (6 electrodes) required 22.5 min.

Although a 5-s interstimulus interval was used during data collection in this study, subsequent tests demonstrated that intervals of ≥ 1 s preserve reliable PRM and MEP detection while substantially accelerating the automated calibration procedure (supplementary material 2), in agreement with previous reports.^28^ In a subsequent study, an interstimulus interval of 2 s was adopted for delivery of the biphasic double pulse, as it was better tolerated by participants than a 1-s interval. This adjustment markedly reduced the total protocol duration to 4.5 min for the midline configuration and 9 min for the bilateral configuration, without compromising reflex elicitation or response detection accuracy.

### B. Identification of the most selective electrode position and stimulation amplitude

After the online detection of spinal reflexes and the near-instantaneous generation of recruitment curves and normalized AUCs for all muscles across each electrode in midline and bilateral configurations, two automated algorithms were developed. The first algorithm, referred to as the **RBA**, is inspired by the work of Salchow-Hömmen *et al.*^27^ and was specifically adapted in this study to evaluate selectivity. This binary method focuses on maximizing either global muscle activation for the conventional approach or the recruitment of specific target muscle groups (proximal or distal on the right or left leg) when selectivity is required. In contrast, the **SDA**, a novel method introduced in this study, is based on quantitative metrics derived from $SI_{T}$ [Eq. (2)] while incorporating the sequential selection criteria described in the **RBA** (Sec. IV D a).

Both algorithms provide an adaptive framework for selecting the optimal electrode position and stimulation amplitude to enhance tSCS selectivity in recruiting specific muscle groups. In the subsequent sections, we present an evaluation of these approaches, analyzing their effectiveness in determining the optimal electrode positions ( $\varepsilon_{p}$) and stimulation amplitude (*Istim*) for different target muscle groups.

Due to limitations related to tSCS tolerance or elevated motor thresholds, data from one participant were excluded for the **midline configuration**, while data from three participants were excluded for the **bilateral configuration**.

#### 1. Comparison between manual single-site selection and automated midline multi-electrode configuration

For conventional single-electrode tSCS, the standard clinical approach, the objective is to activate all lower-limb muscles without selectivity. Under this paradigm, only the **RBA** algorithm was used, as its binary recruitment rule aligns with the goal of identifying the electrode position ( $\varepsilon_{p}$) and stimulation amplitude ( $I_{\text{stim}}$) that maximize global muscle activation.

In the manual method, experienced operators typically select an electrode positioned between T11–T12, corresponding to electrode E2 in our midline configuration. For each participant, the stimulation amplitude is defined as the *first visually identified spinal reflex threshold*, and, in accordance with our protocol, the minimum current for the automated procedure is set to 10 mA below this manual threshold. The manually selected stimulation currents for electrode E2 across the 13 participants were

[20, 35, 20, 40, 30, 35, 40, 30, 30, 35, 30, 30, 30] mA,

representing the consensus threshold determined by the two expert operators.

Using the **RBA** algorithm, however, electrode E2 was selected as the optimal site only for participants S1 ( $I_{\text{stim}}=50$ mA), S2 ( $I_{\text{stim}}=65$ mA), S4 ( $I_{\text{stim}}=56$ mA), S5 ( $I_{\text{stim}}=38$ mA), and S10 ( $I_{\text{stim}}=45$ mA). For the majority of participants, the algorithm selected electrode E3, rather than the manually chosen E2, as the most effective site for global muscle recruitment, including S3 ( $I_{\text{stim}}=27$ mA), S6 ( $I_{\text{stim}}=45$ mA), S7 ( $I_{\text{stim}}=41$ mA), S8 ( $I_{\text{stim}}=40$ mA), S9 ( $I_{\text{stim}}=40$ mA), S11 ( $I_{\text{stim}}=49$ mA), S12 ( $I_{\text{stim}}=29$ mA), and S13 ( $I_{\text{stim}}=40$ mA).

This comparison suggests that the expert-selected electrode (E2) did not consistently achieve maximal global muscle recruitment, either with respect to electrode position or stimulation amplitude, when compared with the proposed method. Based on the automated protocol data, expert selection diverged from the RBA outcome in 8 of the 13 participants, with the RBA identifying the more caudal electrode (E3) and different stimulation currents as producing stronger muscle activation.

Differences in stimulation current between the expert defined threshold and the RBA, selected amplitude were also substantial: among the five participants for whom both methods selected E2 (S1, S2, S4, S5, S10), discrepancies ranged from 8 to 30 mA (e.g., S1 and S2 differed by 30 mA, S4 by 16 mA, S5 by 8 mA, and S10 by 15 mA).

These discrepancies underscore the limitations of manual palpation-based placement and highlight the advantages of automated electrode selection for improving the reliability, consistency, and parameter choice in tSCS calibration.

#### 2. Case of proximal and distal target muscle groups

Table I summarizes the results of RBA and SDA in determining the most selective electrode position ( $\varepsilon_{p}$) along the rostrocaudal axis and stimulation amplitude (*Istim*) to recruit the target proximal or distal muscles groups using **midline configuration**.

**TABLE I.** Electrode position ( $\varepsilon_{p}$) and stimulation amplitude (*Istim*) determined by RBA and SDA, tailored to optimize the activation of proximal or distal muscles (S = 13).

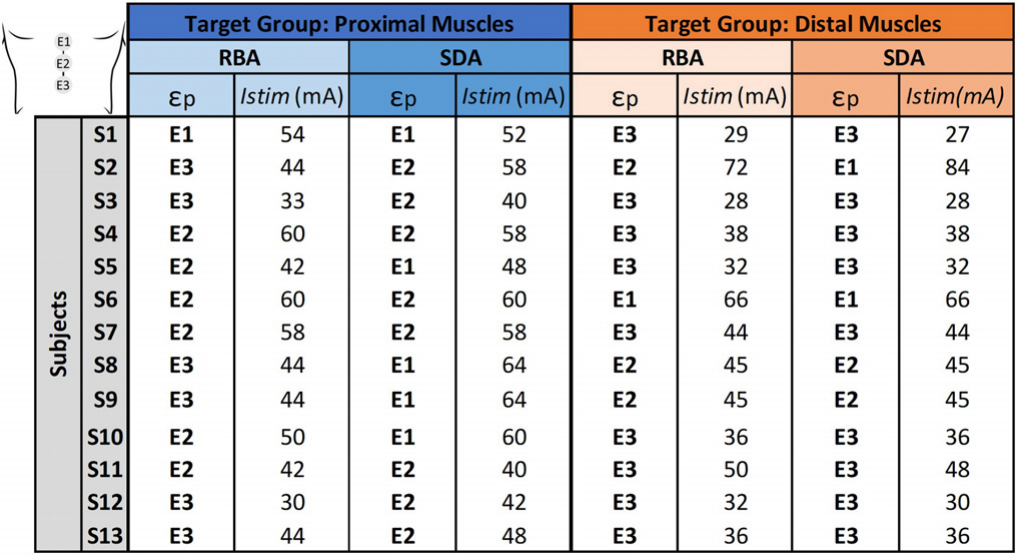

In the group analysis (S = 13), both approaches identified the optimal $\varepsilon_{p}$ and *Istim* for each target muscle group. While similar outcomes were observed between the two methods in certain cases—such as for subjects S4 to S10 when targeting distal muscles and for subjects S6 and S7 when targeting proximal muscles—discrepancies were evident in the selection of the optimal electrode. These differences highlight variations in the underlying criteria used by each algorithm, which will be further discussed in subsequent sections.

#### 3. Case of unilateral proximal, distal, and full-leg target muscle groups

This section presents the outcomes of the RBA and SDA in selecting the optimal spinal electrode and stimulation amplitude using **bilateral configuration**, to enhance the selectivity for proximal and distal muscle groups on both the right and left legs.

In the group analysis (S = 11), regarding ipsilateral selectivity, both algorithms demonstrated a clear tendency to select ipsilateral electrodes (reported in supplementary material 3). When combining for rostrocaudal and ipsilateral selectivity (Table II), both algorithms identified the optimal $\varepsilon_{p}$ and *Istim* to maximize the recruitment of the target proximal or distal muscle groups on the corresponding leg.

**TABLE II.** Electrode position ( $\varepsilon_{p}$) and stimulation amplitude (*Istim*) determined by the RBA and SDA, tailored to optimize the activation of rostrocaudal and ipsilateral target muscle groups (S = 11).

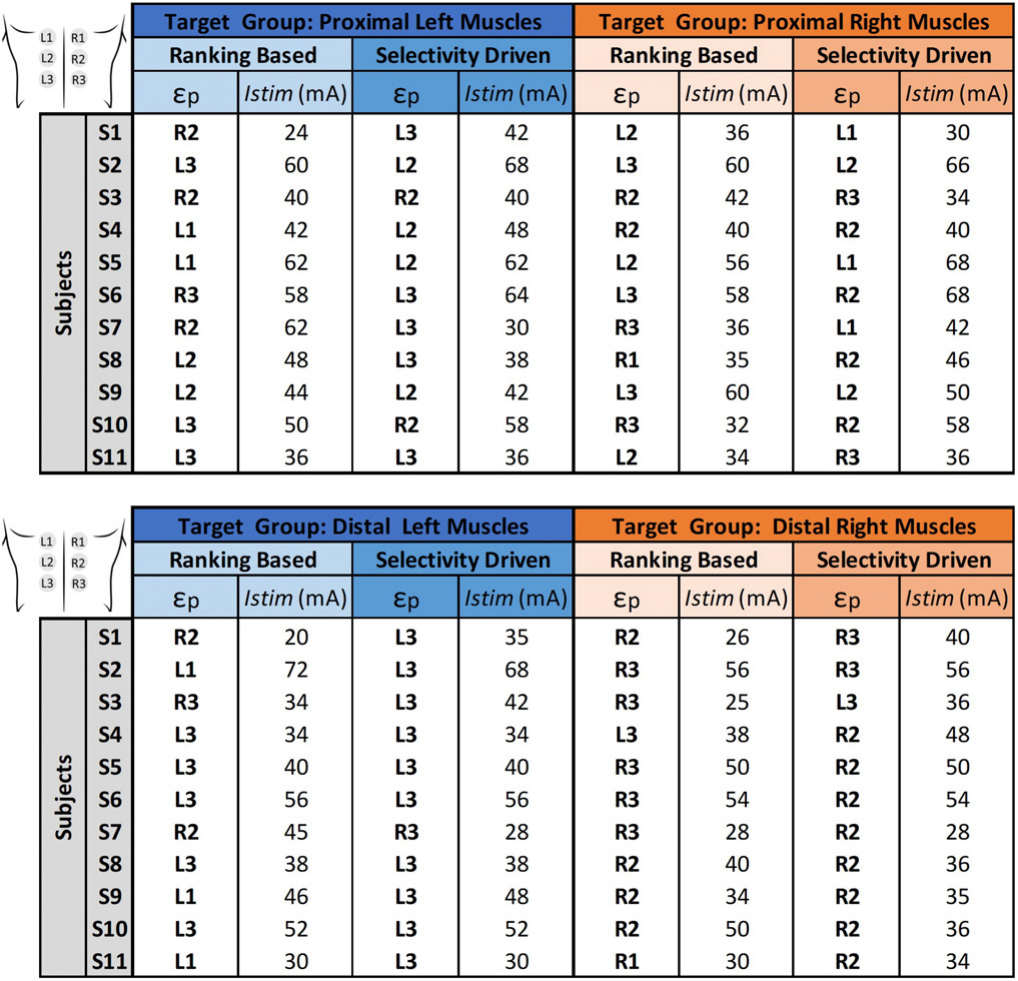

A general trend toward both rostrocaudal and ipsilateral selectivity in electrode selection was observed, aligning with previous findings in the literature. However, discrepancies between the two approaches emerged, particularly when targeting proximal muscle groups, where electrode selection occasionally diverged. Variations were also noted along the rostrocaudal axis and in lateral positioning. The variability in selectivity was especially pronounced when comparing the recruitment of proximal vs distal muscle groups across the legs.

To quantitatively assess how both algorithms behave in the selection of electrode and stimulation parameters, we computed the NRD using offline analysis. The NRD was 0.326 (32.6%) for the **midline configuration** and 0.545 (54.5%) for the **bilateral configuration**, reflecting differences in how each approach identified the electrode with the highest selectivity for a given target muscle group.

### C. Ground truth—comparison between automated algorithm decisions and offline multi-electrode tSCS selectivity

This section presents the offline analysis of the selectivity index for each target muscle group ( $SI_{T}$) across **midline and bilateral configuration**. The analysis aims to: (i) provide robust evidence supporting the selective potential of multi-electrode tSCS, and (ii) compare $SI_{T}$ across different electrode configurations within both setups.

By evaluating $SI_{T}$ for both proximal and distal muscle groups, as well as for target muscles on the right and left legs, this study offers critical insights into the effectiveness of tSCS in achieving selective muscle activation across different electrode configurations. The analysis also underscores the inherent challenges in attaining high selectivity. This comprehensive assessment of $SI_{T}$ under diverse conditions reinforces the pivotal role of electrode configuration and stimulation parameters in optimizing selective tSCS.

#### 1. Rostrocaudal selectivity

In this topic, we investigated muscle recruitment selectivity in response to tSCS using three electrodes positioned from rostral to caudal (E1–E3). This analysis was performed for each electrode using **midline configuration**. Table III presents the $SI_{T}$ corresponding to the positive value representing the strongest selectivity trend for each electrode. Since the analysis involves only two groups, the selectivity values are symmetric (differing only in sign). Therefore, only the positive values are displayed, representing cases where the selectivity favors the target group. The colors represent an $SI_{T}$ scale (shown below in Table III), indicating the electrode's selectivity for proximal muscle groups (blue) and distal muscle groups (orange).

**TABLE III.** Rostrocaudal selectivity index for proximal and distal muscles across subjects and electrode positions. Numbers represent selectivity index values for each target muscle group $SI_{T}$. Color coding follows the $SI_{T}$ scale, indicating each electrode's selectivity—blue for proximal and orange for distal muscle groups.

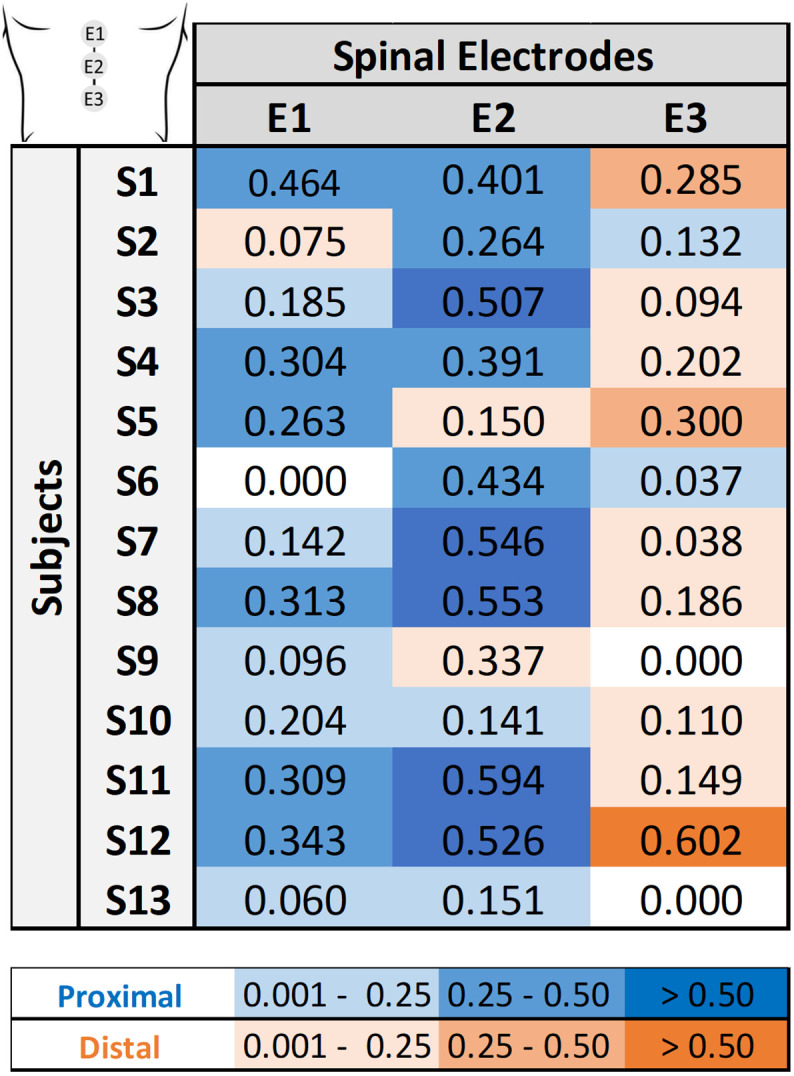	

The results, presented in Table III, reveal a tendency for proximal selectivity in electrodes E1 and E2, with electrode E2 occasionally exhibiting the highest proximal $SI_{T}$. Conversely, electrode E3 tended to bias recruitment toward the distal muscle group in most participants, although selectivity values were modest in several cases. This pattern indicates that E3 showed a directional trend favoring distal rather than proximal recruitment. The low *SI* values, which may be associated with the known limited spatial specificity of tSCS, may also reflect the broad stimulation current range used in this study for research purpose. Selectivity may be enhanced in future work by reducing the stimulation current range or by implementing real-time models, for example, AI-driven tSCS methods.

In the group analysis ( S=13), for the proximal and distal muscles [Fig. 2(a)], the Kruskal–Wallis test revealed significant differences between the electrodes ( H=18.3261, p=0.0001), demonstrating that electrode position influences the selectivity of activation. Post-hoc comparisons using the Wilcoxon test corrected with Benjamini–Hochberg further clarified these findings. The difference between E1 (the most rostral electrode) and E2 was not significant ( p=0.1909), while both E1 and E2 showed significant differences relative to E3 (the most caudal electrode), with the Benjamini–Hochberg-corrected pairwise comparison yielding p=0.0051.These results suggest that the caudal electrode (E3) promotes greater selectivity for distal muscles compared to the rostral electrodes (E1 and E2), which, in turn, exhibit a higher selectivity for the proximal muscle group.

#### 2. Ipsilateral selectivity

In this analysis, we investigated the ipsilateral selectivity of tSCS, focusing exclusively on comparisons between the electrodes on the right (R1, R2, and R3) and the left (L1, L2, and L3), corresponding to the muscle groups of the right and left legs, respectively. This analysis was performed for each electrode using **bilateral configuration**. Here, we do not show the analyses of differences between electrodes on the same side, as the primary objective is to evaluate ipsilateral selectivity.

For most subjects, the $SI_{T}$ of the spinal electrodes L1, L2, and L3 is higher for the left leg muscles, while the spinal electrodes R1, R2, and R3 exhibit greater selectivity for the right leg muscles. However, despite the presence of at least one electrode per subject displaying ipsilateral selectivity—except for subject S10, who shows left leg selectivity only in electrode R2—it is notable that several cases reveal a contralateral selectivity pattern. This is particularly more evident for electrode L1, which demonstrates higher selectivity for contralateral muscles in several subjects (S1, S5–S8, and S10). Supplementary material 4 reports detailed ipsilateral selectivity metrics per-subject $SI_{T}$.

In the group analysis ( S=11), the Kruskal–Wallis test revealed significant differences in selectivity for both the left and right leg muscle groups ( H=29.2674, p<0.0001), demonstrating a strong ipsilateral selectivity pattern. Post hoc comparisons showed that electrodes on the same side of the spinal cord as the targeted leg exhibited significantly higher selectivity compared to contralateral electrodes. For the left leg, significant differences were observed in pairs such as L2 vs R1 ( p=0.0293), L2 vs R2 ( p=0.0073), and L2 vs R3 ( p=0.0059), with L3 showing stronger ipsilateral selectivity compared to R1 ( p=0.0049), R2 ( p=0.0059), and R3 ( p=0.0049). Similarly, for the right leg, ipsilateral selectivity was evidenced by significant differences in pairs such as R3 vs L1 ( p=0.0483), R3 vs L2 ( p=0.0059), and R3 vs L3 ( p=0.0049), with R2 and R1 also demonstrating higher selectivity compared to L3 ( p=0.0059 and p=0.0049, respectively).

These results reinforce the predominance of ipsilateral selectivity,^25^ where stimulation applied to one side of the spinal cord predominantly activates the muscles of the corresponding leg. The observed differences between the contralateral electrodes highlight that the effects of stimulation are localized and highly influenced by the lateralization of the applied stimulus. Moreover, the findings suggest distinct selectivity patterns along the rostrocaudal axis, with the more caudal electrodes (L3, R3) exhibiting greater selectivity for ipsilateral leg muscles.

#### 3. Rostrocaudal and ipsilateral selectivity

In this analysis, we evaluated the combined rostrocaudal and ipsilateral selectivity of multi-electrode tSCS. In the analysis of the target muscle groups, Table IV presents the $SI_{T}$, corresponding to the positive value that represents the strongest selectivity trend for each electrode. The colors associated with each target muscle group are shown in the legend, highlighting instances where selectivity favors the respective target group.

**TABLE IV.** Rostrocaudal and ipsilateral selectivity index for proximal and distal muscle groups in the right and left legs, across subjects and electrode positions ( S=11). Numbers represent selectivity index values for each target muscle group $SI_{T}$. Color coding represents muscle group and laterality: blue for proximal left, orange for distal left, green for proximal right, and yellow for distal right muscles.

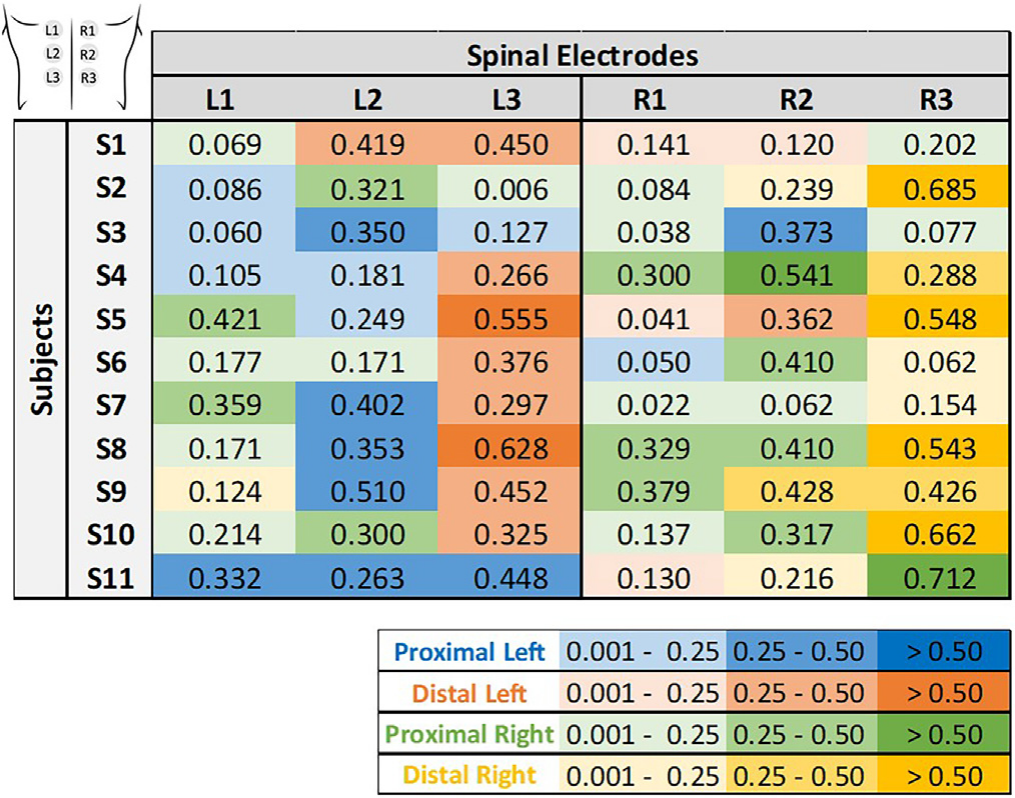

For most subjects, electrode L3 shows stronger evidence of a higher $SI_{T}$ for the distal left muscle group, while electrode R3 demonstrates the highest $SI_{T}$ for the distal right muscle group. Although less evident for proximal muscles, a similar trend can be observed, with rostral electrodes favoring recruitment of proximal muscles in the left leg (L1 and L2) and the right leg (R1 and R2). These findings underscore the combined rostrocaudal and ipsilateral selectivity patterns. However, it is also clear that there is considerable variability between subjects and electrodes, highlighting the complexity of achieving optimal selectivity, a topic further explored in the Secs. III A and III B.

In the group analysis ( S=11), significant differences were identified in the selectivity for the distal left leg muscle group ( H=22.8054, p=0.0004) using the Kruskal–Wallis test. Post hoc comparisons revealed that selectivity is influenced by both rostrocaudal positioning and laterality. Specifically, the most caudal electrode on the left (L3) exhibited significantly greater selectivity compared to the most rostral electrode on the left (L1) ( p=0.0073). Additionally, L3 differed significantly from the right electrodes R1 ( p=0.0073), R2 ( p=0.0073), and R3 ( p=0.0088). These findings suggest that selectivity for distal muscles on the left leg is predominantly associated with caudal electrodes, with minimal influence from contralateral electrodes.

Similarly, for the distal right leg muscle group, exemplified in Fig. 2(b), the Kruskal–Wallis test identified significant differences ( H=29.3099, p<0.0001). Post hoc analysis revealed distinct patterns of selectivity. Electrode R3 showed significantly higher selectivity compared to L1 ( p=0.0037), indicating that the most caudal electrode on the right promotes greater selectivity for ipsilateral distal muscles. Electrode L2 exhibited lower selectivity compared to R1 ( p=0.0049), R2 ( p=0.0458), and R3 ( p=0.0037). Among the right electrodes, R3 demonstrated significantly greater selectivity than R1 ( p=0.0037) and R2 ( p=0.0049). These results emphasize the predominance of ipsilateral selectivity and caudal electrodes in optimizing activation for distal muscle groups.

For the proximal left leg muscles, the Kruskal–Wallis test revealed significant differences ( H=23.8953, p=0.0002), with post hoc comparisons showing distinct patterns of selectivity. Electrode L1 demonstrated significantly higher selectivity compared to R3 ( p=0.0183), while L2 exhibited higher selectivity than R1 ( p=0.0146), R2 ( p=0.0293), and R3 ( p=0.0146). In contrast, L3 showed significant differences only with R3 ( p=0.0205), and no significant differences were observed between L1 and L3 or between L3 and R1 or R2. These findings indicate that ipsilateral selectivity for proximal muscles in the left leg is more pronounced in rostral electrodes, particularly L2.

For the proximal right leg muscles, the Kruskal–Wallis test also indicated significant differences ( H=20.4953, p=0.0010), with post hoc comparisons revealing notable selectivity patterns. Electrode L3 showed significantly lower selectivity compared to R1 ( p=0.0098), R2 ( p=0.0110), and R3 ( p=0.0146). Additionally, L1 and L2 both exhibited lower selectivity compared to L3 ( p=0.0073) but did not significantly differ from the right-side electrodes. Among the right-side electrodes, no significant differences were observed between R1, R2, and R3. These results emphasize that, for the proximal right leg muscles, caudal electrodes such as L3 demonstrate reduced selectivity compared to their rostral counterparts, particularly R1 and R2.

In summary, the findings demonstrate that electrodes positioned more rostrally along the rostrocaudal axis, such as L1 and L2 for the left leg and R1 and R2 for the right leg, exhibit a marked tendency to selectively recruit unilateral proximal muscle groups. Conversely, and more prominently, the electrodes L3 for the left leg and R3 for the right leg show a strong tendency to selectively recruit unilateral distal muscle groups. This pattern underscores the critical role of electrode positioning in optimizing the activation of specific muscle groups, particularly proximal muscles, through lateralized and segmental stimulation mechanisms.

## III. DISCUSSION

This study aimed to improve the selectivity of tSCS using multi-electrode configurations and to implement automated, online methods that personalize stimulation parameters. These approaches enable the selective activation of target muscle groups and support the clinical translation of tSCS for gait rehabilitation. In a cohort of 14 participants, we developed an online tSCS framework capable of detecting spinal reflexes and EMG-based muscle responses online. The online detection was validated against offline analysis, showing consistent recruitment curves and synchronization with EMG recordings.

Our findings reinforce the evidence of rostrocaudal and ipsilateral selectivity in tSCS: rostral electrodes predominantly activated proximal muscles, whereas caudal electrodes favored distal muscle recruitment. Lateral configurations further enhanced side-specific activation. However, substantial variability was observed across participants and electrode sites, highlighting the inherent challenges in achieving consistent selectivity due to anatomical variability, electrode placement, and inter-subject differences. These findings underscore the potential of automated, online algorithms to optimize both single- and multi-electrode tSCS protocols.

To address this, we introduced two novel algorithms designed to optimize electrode positioning and stimulation amplitude in multi-electrode tSCS. These methods aim to enhance the recruitment specificity of target muscle groups and overcome key limitations of conventional single-electrode stimulation.

The Secs. III A and III B present a detailed analysis of these findings and their implications for targeted neuromodulation and neurorehabilitation.

### A. Automated algorithms optimize electrode position and amplitude to enhance tSCS selectivity for targeted muscle group recruitment

To our knowledge, this is the first study to develop a framework for online spinal reflex detection using PRM test and automated algorithms to optimize muscle selectivity through a multi-electrode tSCS approach.

The proposed online and automated protocol leverages EMG-based detection of spinal reflexes via PRM testing to accelerate stimulation procedures and enable more efficient analysis of muscle selectivity. Although Spieker *et al.*^29^ recently introduced a machine learning-based method using mechanomyography (through inertial measurement units, IMUs) to automate spinal reflex detection, their approach primarily aims to streamline the setup and reduce the testing time. In contrast, although EMG-based protocols, such as those used in this study and by Salchow-Hömmen *et al.*,^27^ may involve longer setup times, EMG provides a more accurate and reliable measure of muscle activation, which is critical for detailed selectivity analysis.

• **High-accuracy online spinal reflex detection can facilitate clinical translation of single- and multi-electrode tSCS and improve calibration efficiency**

The comparison between online and offline classifications shows that the proposed online spinal reflex detector is reliable for real-time multi-electrode calibration, achieving an accuracy of 87%. Most misclassifications resulted from imprecise artifact localization rather than incorrect interpretation of the physiological response, as confirmed by offline analysis.

Importantly, the method uses the same empirically defined 70% suppression threshold validated offline, demonstrating that a single non-adaptive criterion is adequate for stable reflex identification across participants. This threshold, however, may require validation and possible adjustment in future studies involving individuals with SCI, although prior work suggests it is appropriate for reliable suppression-based classification.^27^

This framework constitutes a methodological advancement, since reliable online identification of proprioceptive-afferent recruitment is essential for enabling data-driven optimization of electrode placement and stimulation intensity according to muscle-group selectivity. By providing reflex-based feedback during the acquisition process, this tool lays the groundwork for future developments such as AI-driven real-time optimization models capable of dynamically adapting the stimulation parameters. Overall, these results demonstrate the feasibility and translational potential of incorporating online spinal reflex detection into tSCS workflows to enhance the precision and efficiency of neuromodulation protocols.

• **Manual expert-based electrode and amplitude selection diverges from data-driven algorithmic outcomes, potentially limiting the efficacy of conventional single-site tSCS**

Although trained operators selected the midline electrode at the T11–T12 level (E2) using palpation and defined the stimulation intensity based on their experience and on the first visually identified spinal reflex, as is standard practice in clinical tSCS protocols, this approach did not reliably recruit all muscles when compared against the full dataset and the selection obtained with the RBA algorithm.

The automated RBA method diverged from the expert-defined placement in 8 of the 13 participants, most often identifying the more caudal electrode (E3) as producing stronger recruitment across all lower-limb muscles. Differences in stimulation current between the expert-defined threshold and the RBA-selected amplitude were also substantial. Among the five participants for whom both methods selected E2 (S1, S2, S4, S5, and S10), discrepancies ranged from 8 to 30 mA. These differences exceed the ranges reported by Salchow-Hömmen *et al.*,^27^ who observed that stimulation amplitudes selected by their automated approach typically differed by only 5 to 10 mA from expert-defined settings across 15 participants (healthy controls, individuals with SCI and Parkinson's disease). This comparison further highlights that manual amplitude estimation can vary considerably even among trained operators.

It is also important to note that when moving from conventional single-electrode stimulation to multi-electrode configurations and selectivity analysis, manual calibration becomes nearly impractical and insufficiently reliable, even for trained operators. Quantifying the relative activation of one muscle group vs another, and determining which electrode position and stimulation amplitude maximize selective recruitment, are tasks that exceed what can be assessed visually or by clinical experience alone. For this reason, a direct comparison between manual and algorithmic selection was not performed for the multi-electrode selectivity scenarios, as manual methods cannot provide an objective or consistent benchmark for selective recruitment.

Collectively, these findings underscore that palpation-based electrode placement and visually guided reflex thresholding do not necessarily correspond to the stimulation parameters that produce the strongest or most reliable global recruitment. Instead, operator-dependent variability in both electrode placement and threshold identification can lead to suboptimal calibration, an issue that may compromise the efficacy of tSCS in clinical rehabilitation. Furthermore, this dependence on specialized operators poses an additional barrier to clinical translation and broad usability, particularly for multi-electrode tSCS approaches that require consistent and precise electrode placement.

By contrast, the automated algorithms offer a standardized, data-driven alternative that minimizes subjective variability, improve consistency across participants, and provide a more individualized stimulation prescription. These advantages are particularly relevant for clinical workflows, in which time efficiency, reproducibility, and parameter precision are essential for maximizing therapeutic outcomes.

• **Multi-electrode tSCS Enables Rostrocaudal and Ipsilateral Selectivity but is Limited by Inter-Subject Biophysical and Anatomical Variability**

Multi-electrode tSCS can generate rostrocaudal and ipsilateral biases in muscle-group recruitment, consistent with prior findings.^23,25,26^ In the midline configuration, electrodes E1–E2 generally favored proximal activation, whereas E3 tended to bias recruitment toward distal muscles. In the bilateral configuration, combined rostrocaudal–ipsilateral trends were observed; however, occasional contralateral activation occurred, likely reflecting anatomical asymmetries and individual differences in spinal alignment that influence current distribution.^14,30,31^

Electrode placement variability is another major contributor to inconsistent selectivity. Manual palpation of vertebral landmarks can vary substantially—up to 23% across individuals^26,32^ and similar variability was observed in our dataset. Such imprecision, combined with biophysical factors affecting current spread^30^) likely explains much of the heterogeneity across participants.

Overall, these findings reinforce that while multi-electrode tSCS is capable of producing meaningful directional selectivity, the magnitude and consistency of this selectivity remain constrained by (i) anatomical variability;^30,33^ (ii) imprecise electrode placement inherent to manual techniques;^27^ and (iii) biophysical factors affecting current spread, such as variations in vertebral structure, skin, and adipose tissue.^30^

Together, these factors highlight the limitations of manual approaches and the critical role of electrode placement and stimulation parameters in achieving reliable selectivity. In this context, the algorithms proposed here show promise for overcoming these limitations and open new avenues for clinical applications by enabling multi-electrode tSCS tailored to individual recruitment profiles and electrode configurations.

• **Automated spinal reflex detection and selectivity-driven tSCS algorithms enhance muscle-group recruitment while reducing variability and reliance on specialized operators.**

Manual single- and multi-electrode tSCS calibration is inherently time-consuming and highly dependent on the clinician's experience.^26,27^ Although prior studies do not report explicit setup times, both Salchow-Hömen *et al.* and Bryson *et al.* highlight factors that directly prolong calibration. Palpation-based electrode placement is imprecise: vertebral count variations occur in 2%–23% of the population, and both studies report that manual repositioning of electrodes is frequently required when desired muscle responses are not obtained on the first attempt.^26,27^ In Bryson *et al.*, repositioning was required in five participants, illustrating that conventional placement often demands several iterations before reliable recruitment is obtained. These findings suggest that manual electrode placement rarely succeeds on the first attempt and may significantly prolong setup time and reduce the efficacy of tSCS for gait rehabilitation.

With the stimulation parameters used in this experiment (5-s interstimulus interval), the automated approaches proposed here did not confirm our initial hypothesis of accelerating tSCS calibration when compared with the conventional single-electrode method, which typically requires approximately 10 min.^27^ When comparing multi-electrode approaches, manual setup required 15 and 25 min for the midline and bilateral configurations, respectively, whereas automated calibration required 11.25 and 22.5 min for the same configurations. Importantly, the primary objective of the proposed framework is not solely time reduction, but rather the optimization of stimulation selectivity. Achieving this goal necessarily requires systematic exploration of multiple electrodes and stimulation amplitudes to identify the most selective electrode location and optimal current intensity.

Manual assessment of muscle-group selectivity is also impractical within clinical workflows. Differentiation between proximal and distal or left and right muscle recruitment is typically performed visually, making selectivity difficult to quantify and substantially increasing operator burden. In contrast, algorithmic processing enables immediate and numerical computation of selectivity metrics, allowing for objective and reproducible assessment of muscle recruitment patterns while reducing dependence on operator expertise.

Following data collection for this study, further protocol refinements were identified that substantially reduce calibration duration. Specifically, reducing the interstimulus interval from 5 to 2 s decreased the total protocol time to 4.5 min for the Midline configuration and 9 min for the Bilateral configuration, without affecting PRM or MEP detection. Calibration time could be reduced even further. Although the present protocol employed 15 stimulation steps with 2 mA increments for research purpose, the same stimulation range could be covered using 5 mA increments, requiring only six steps. This modification would reduce the duration of automated calibration by nearly a factor of three and would also shorten calibration time when compared to the conventional single-electrode approach.

Overall, these results demonstrate that the proposed automated multi-electrode tSCS framework enhances selective targeting of muscle groups while substantially reducing operator-dependent expertise and the need for repeated electrode repositioning—three major barriers to effective clinical translation of tSCS.^27^ In addition, the framework shows potential to reduce overall setup time, even when compared with single-electrode placement, while simultaneously providing optimized stimulation parameters. Such automation may improve accessibility, reliability, and reproducibility in clinical environments by minimizing errors associated with manual electrode placement and inter-individual anatomical variability.

• **SDA Favors Selective Recruitment—RBA Suits Rapid and Generalized Applications in Single-Electrode tSCS**

We developed two algorithms—the **RBA** and the **SDA**—to determine the optimal electrode position and stimulation amplitude for maximizing the recruitment of target muscle groups. Compared to offline $SI_{T}$ ground truth analysis, both algorithms identified optimal electrode positioning and stimulation amplitude for selective activation of the proximal or distal muscles in either leg. Although additional testing across multiple sessions is required to assess robustness, we suggest that **SDA** is a more reliable approach for achieving higher selectivity. In contrast to the **RBA**, which uses binary reflex detection, the SDA defines optimal stimulation parameters based on the quantitative assessment of muscle activation to enhance selectivity.

Compared with offline $SI_{T}$ as ground truth, our findings indicate that both algorithms identified optimal parameters across all targets in both the **midline** and **bilateral** configurations. The results align with the trends of the rostrocaudal and ipsilateral selectivity patterns, reflecting the anatomical and functional organization of the spinal cord,^23,26,34^ while also demonstrating the ability to account for deviations due to spinal variability by adapting to individual recruitment profiles and electrode configurations.

Although both algorithms occasionally converged on the same electrode and stimulation amplitude, notable discrepancies emerged in scenarios involving more complex selectivity demands. These divergences provide insight into the distinct decision-making strategies employed by each algorithm and their respective sensitivities to recruitment patterns.

Each approach contributes to distinct strengths and applications. The **RBA** is well-suited for rapid, generalized applications. Its binary classification enhances the conventional single-electrode tSCS approach by providing an automated method to identify the electrode that maximizes overall muscle activation within the **midline configuration**. Our findings indicate a preference for caudal electrodes (E2–E3), in alignment with the anatomical enlargement of the lumbosacral region.^23,26,34^ Although it can also be applied to target specific muscles, the simplicity of this approach may limit its capacity to refine selectivity, adapt to inter-subject variability, or achieve highly accurate recruitment of distinct motor groups.

We suggest that the **SDA** is more suitable for selectively recruiting target muscle groups. By integrating quantitative metrics derived from ( $SI_{T}$), it optimizes electrode placement and stimulation amplitude for the recruitment of proximal, distal, and unilateral targets. It systematically compares activation values across muscles and stimulation amplitudes, addressing complex selectivity requirements.

The output of both algorithms was validated against the offline $SI_{T}$ analyses. While in some cases both approaches converged to similar outcomes, individual analyses revealed that the **SDA** aligned more consistently with offline selectivity indices, often identifying electrodes with higher $SI_{T}$. For instance, in the midline configuration, the **SDA** identified E2 as the most selective electrode, whereas the **RBA** selected E3 for subject S2. In **bilateral configuration**, where selectivity patterns are more complex, the **SDA** also shows higher accuracy relative to $SI_{T}$, underscoring its value for advanced stimulation paradigms.

Since lower stimulation amplitudes enhance selectivity and electrode positioning critically shape recruitment patterns, alternative strategies beyond AUC-based metrics^30^ and binary classification^27^ are essential. In this context, our findings underscore the complementary value of automated algorithms for multi-electrode tSCS. The **RBA** provides a fast and generalized solution for global activation, while the **SDA** provides refined selectivity for targeted applications. Both approaches hold promise for clinical translation, facilitating reliable muscle recruitment for locomotor tasks and rehabilitation.

### B. Potential of multi-electrode tSCS, clinical integration, and limitations

Current clinical studies primarily use single-electrode tSCS with fixed parameters to achieve generalized lower-limb muscle recruitment. This approach has shown promise for SCI rehabilitation, benefiting lower limbs.^35^ Reported improvements include reduced spasticity,^36^ enhanced voluntary gait,^19,20,35,37–39^ and better trunk stability.^40^

Recently, tSCS has been explored for other motor impairments, including cerebral palsy (CP)^41–44^ and stroke.^45,46^ Given the diverse motor deficits in these conditions, we hypothesize that multi-electrode tSCS during gait training could enhance rehabilitation by enabling targeted muscle activation and task-specific neuromodulation.

This concept aligns with findings from invasive eSCS, where multi-electrode stimulation enhances control over joint movements, improving functions like standing, walking, cycling, and swimming.^47^ A multi-electrode tSCS approach could similarly refine neuromodulation strategies, offering more selective and dynamic gait rehabilitation.

However, our findings reveal inherent challenges in achieving consistent selectivity due to anatomical variability, electrode placement, and inter-subject differences. While the **SDA** mitigates some of these issues, further refinements are needed to enhance robustness and reliability. A key advancement could be real-time optimization algorithms that dynamically adjust stimulation parameters based on immediate feedback. Artificial intelligence-driven models, as proposed for eSCS,^48,49^ could optimize tSCS strategies by adapting electrode selection and stimulation amplitudes during therapy.

Another improvement involves increasing spinal electrode density,^24,50^ allowing finer control over stimulation sites and optimizing muscle recruitment. Further research is needed to determine the ideal electrode configuration for both efficacy and patient comfort.

This study introduces and validates the **RBA** and **SDA** to optimize tSCS selectivity. Real-time feedback and expanding electrode arrays could further improve clinical outcomes, paving the way for more precise and personalized neurorehabilitation.

## IV. METHODS

### A. Participants and experimental setup

Fourteen neurologically intact participants (five males and nine females; age: 26.0 ± 5 years, height: 177.6 ± 13 cm, body mass: 75.0 ± 45.0 kg) were recruited for this study. Muscle activity was recorded using surface EMG from four proximal muscles: rectus femoris (RF), semitendinosus (ST), vastus lateralis (VL), and vastus medialis (VM) and three distal muscles: gastrocnemius (GASTRO), tibialis anterior (TA), and soleus (SOL) on both legs. EMG signals were captured using the Trigno Research+ system (Delsys, USA), with electrode placement following the Surface Electromyography for the Non-invasive Assessment of Muscles (SENIAM) guidelines reference.^51^

tSCS was delivered over the lumbosacral spinal cord, which has been shown to facilitate synaptic transmission and activate motor neuron pools innervating lower limb muscles.^19^ Stimulation electrodes (Axelgaard, Denmark) were placed over the lower thoracic (T10–T12) and upper lumbar (L1) vertebrae, based on anatomical maps and the segmental organization of motor pools within the lumbosacral enlargement of the spinal segments L1–L5 and S1–S2.^34,52–54^ Selectivity was analyzed concerning both rostrocaudal and ipsilateral recruitment, as determined by the spatial arrangement of the stimulation electrodes.

Muscles were grouped according to the specific electrode configuration and stimulation targets:

• **Midline Configuration**: Three 2.5 cm self-adhesive surface electrodes were placed along the spinal midline at the T10–T11, T11–T12, and T12–L1 vertebral levels (Fig. 1-1). This configuration targeted three muscle groups: proximal, distal, and all lower limb muscles.
• **Bilateral Configuration**: Six 2.5 cm self-adhesive electrodes were positioned bilaterally—three on each side—2.5 cm lateral to the midline, spanning the T10–L1 spinous processes (Fig. 1-1). This configuration targeted six muscle groups: proximal left, proximal right, distal left, distal right, all right–leg muscles, and all left-leg muscles.

The choice to analyze muscle groups rather than individual muscles is justified by the limited ability of tSCS to selectively recruit single muscles, as demonstrated by Bryson *et al.,*^26^ and are further supported by our own results, presented in supplementary material 1. Moreover, this study does not distinguish between agonist and antagonist muscle groups, as current evidence does not clearly elucidate the mechanisms underlying selective activation of flexor vs extensor muscles. Nevertheless, findings from eSCS studies suggest that higher stimulation frequencies preferentially recruit flexors, whereas lower frequencies tend to activate extensors—likely reflecting the polysynaptic vs monosynaptic organization of these pathways—which may enhance coordination and functional gait patterns.^6,9^ These frequency-dependent recruitment properties may also represent a promising direction for future tSCS studies.

Two large electrodes (8 × 13 cm) were positioned paraumbilically and used as bilateral reference electrodes, activated simultaneously across all stimulation protocols. Stimulation was delivered using NeuroPulse, a non-invasive eight-channel electrical stimulator developed at the REHAssist Laboratory, EPFL, Switzerland. This highly versatile device delivers charge-balanced biphasic pulses with fully adjustable parameters (frequency, pulse width, amplitude, and carrier frequency), enabling multi-electrode tSCS with automated parameter adjustments. Its design allows for a common paraumbilical anode to be shared between all cathodes positioned over the spinal cord.

Charge-balanced biphasic double pulses were applied. Each pulse had a 1 ms width with a 50 ms interpulse interval. During stimulation, the spinal electrodes acted as the anode in the first phase and the cathode in the second, relative to the paraumbilical electrodes. This anode-leading, charge-balanced biphasic configuration follows established multi-electrode tSCS protocols reported in prior studies.^26,27^ The electrode positions at the T10–T11, T11–T12, and T12–L1 vertebral levels were identified through manual palpation, which was independently validated by a second researcher from the group. This process involved first locating the iliac crest and aligning it with the L4 spinous process as a reference point. Subsequently, the manual palpation method was applied to accurately identify the targeted vertebrae.

### B. Stimulation loop protocol

To evaluate tSCS selectivity, participants underwent the PRM reflex test in the supine position using both midline and bilateral electrode configurations. An automated online protocol for spinal reflex detection was developed (Sec. IV B), implemented in Python, and integrated with the Delsys API to ensure synchronized stimulation delivery and EMG data acquisition. During stimulation, muscle activity was continuously monitored.

First, subject-specific stimulation current ranges were established for each electrode configuration using the PRM reflex test. The minimum current was set to 10 mA below the visually identified reflex threshold, providing a conservative baseline, while the maximum current corresponded to the highest intensity tolerated by the participant. The visually identified reflex threshold was also used as the reference current that would be selected during a conventional manual procedure.

For each electrode configuration, stimulation was delivered sequentially across the defined current range. At each amplitude, three stimulation pulses were applied with a 5-s inter-stimulus interval. The current was initially increased in 5 mA steps. Upon online detection of the first spinal reflex, the increment was automatically reduced to 2 mA to improve resolution. Subsequently, fifteen additional stimulation amplitudes were delivered in 2 mA steps, up to either 30 mA above the reflex threshold or the participant's maximum tolerable intensity.

#### 1. Online Spinal reflex detection

An automated pipeline was developed for online spinal reflex detection during tSCS, based on synchronized EMG acquisition and stimulation timing. Online synchronization between stimulation events and EMG recordings was achieved by segmenting the data into 5-s windows for each repetition, using stimulation artifacts as temporal markers to ensure accurate alignment between stimulation pulses and the corresponding EMG signals.

EMG signals were pre-processed to remove baseline offsets and minimize noise interference. First, the median value and identified artifacts were removed. A second-order Butterworth notch filter (48–53 Hz) was applied to suppress power-line interference, followed by a first-order Butterworth low-pass filter with a 300 Hz cutoff to attenuate high-frequency components.

The noise level for each muscle, stimulation amplitude, repetition, and electrode was calculated as the standard deviation of the signal during the resting period ( 2.5 s<t<5 s). This calculated noise threshold was used to detect signal features that exceeded baseline noise.

To identify stimulation artifacts used for EMG–stimulation synchronization, a non-causal double differentiation was applied to the raw EMG signals using a discrete approximation of the Laplacian operator ( Δ). The resulting processed signal for each muscle *i* was computed as

$${\text{emg}}_{i,\Delta }(E,\text{AMP},j,\tilde{t})=4\cdot \Delta \left({\text{emg}}_{i,\text{raw}}(E,\text{AMP},j,\tilde{t})\right),$$ (1)

where *i* is the muscle index, $\tilde{t}\in [0.8\;s,1.25\;s]$ defines the time window of interest, E∈{1,2,…,6} is the electrode position, *AMP* is the stimulation amplitude, and *j* is the repetition index. Candidate artifact peaks ${\tilde{t}}^{*}$, corresponding to the first stimulation pulse, were detected if all of the following conditions were met:

• **Criteria 1:** The peak amplitude exceeded three standard deviations ( 3 SD) of the baseline noise:
$$\left|{\text{emg}}_{i,\Delta }(E,\text{AMP},j,{\tilde{t}}^{*})\right|>3\cdot \text{SD}\left({\text{emg}}_{i,\text{noise},\Delta }\right).$$ (2)
• **Criteria 2:** A second peak at ${\tilde{t}}^{*}+50$ samples (interpulse interval) also exceeded this threshold:
$$\left|{\text{emg}}_{i,\Delta }(E,\text{AMP},j,{\tilde{t}}^{*}+50)\right|>3\cdot \text{SD}\left({\text{emg}}_{i,\text{noise},\Delta }\right).$$ (3)
• **Criteria 3:** The two peaks showed comparable amplitudes, defined by
$$1-\frac{\text{min}\left\{p2p({\tilde{t}}^{*}),p2p({\tilde{t}}^{*}+50)\right\}}{\text{max}\left\{\text{amp}({\tilde{t}}^{*}),\text{amp}({\tilde{t}}^{*}+50)\right\}}<0.8.$$ (4)

Here, $p2p({\tilde{t}}^{*})$ and $p2p({\tilde{t}}^{*}+50)$ represent the peak-to-peak amplitudes within a 2 ms window centered at each respective peak. Trials meeting all three criteria were labeled as stimulation artifacts. The timestamp of the first detected artifact ( $t_{0}$) was then used to synchronize the EMG signals across all electrodes, muscles, and trials.

#### 2. Automatic evaluation of spinal reflex

In the detection of motor evoked potentials (MEPs), peaks exceeding the noise threshold ( $3\cdot \text{SD}\cdot emg_{i,\text{noise}}$) are identified within the time window $t_{0}<t<t_{0}+40\text{ ms}$. When a peak is detected, its timestamp is recorded as $t_{\text{max}}$, and peak-to-peak amplitudes are calculated for two intervals: $t_{\text{max}}-8\text{ ms}<t<t_{\text{max}}+15\text{ ms}$ and $t_{\text{max}}+42\text{ ms}<t<t_{\text{max}}+65\text{ ms}$. These amplitudes are denoted as $amp_{1,i}(n,I)$ and $amp_{2,i}(n,I)$, representing the first and second response amplitudes, respectively.

The suppression level of the second response relative to the first is then computed to evaluate the signal's characteristics. A response is classified as a MEP if it meets the following criteria:

• The suppression level of the second response exceeds 70%. While most studies classify spinal reflexes based solely on an amplitude ratio $R_{2}/R_{1}<1$,^26,52,55^ we adopted a stricter suppression-based criterion, consistent with the online 50% threshold-based approach proposed by Salchow-Hömen *et al.*,^27^ in order to minimize false detections during automated and online processing.
• The first response amplitude, $amp_{1,i}(n,I)$, is greater than 50 μV.
• The first response amplitude also surpasses the threshold of $3\cdot \text{SD}\cdot emg_{i,\text{noise}}$.

These thresholds ensure that detected MEPs are both physiologically relevant and distinguishable from noise.

Evoked responses for each muscle were normalized relative to the average maximum evoked response recorded across the three electrodes in **midline configuration** or the six electrodes in **bilateral configuration**. This normalization was performed independently for each condition to account for variations in maximum response amplitudes. The normalization process generated recruitment curves that represent the percentage of muscle activation associated with each electrode configuration, providing a detailed assessment of activation patterns and selectivity.

### C. tSCS selectivity analysis

Recruitment curves were constructed for each muscle using data obtained from the automated stimulation protocol, considering **midline configuration** and the **bilateral configuration**. At each electrode position and stimulation amplitude, three repetitions of double-pulse stimulation were delivered. The average peak-to-peak amplitude of the first pulse was computed across repetitions that elicited a detectable spinal reflex.

For each muscle, the evoked responses were normalized relative to the highest average response recorded across all electrodes within each electrode configuration. In cases where a response was absent at a given stimulation amplitude but present at the previous amplitude, the last valid response value was carried forward to maintain continuity in the recruitment curve and minimize false negatives. Muscle recruitment was then quantified by calculating the area under curve (AUC), representing the cumulative response of each muscle over the range of stimulation intensities. These AUC values were subsequently normalized by the maximum observed recruitment value, yielding the normalized recruitment metric, denoted as *REC*.

The selectivity index (*SI*)^26^ was calculated to quantify how selectively a specific muscle is recruited, by comparing its normalized activation to the average activation of all other muscles. Mathematically, the *SI* for muscle *m* is defined as

$$SI_{m}=REC_{m}-\frac{\sum_{n\neq m}^{M}REC_{n}}{M-1},$$ (5)

where $REC_{m}$ denotes the normalized recruitment (AUC) of muscle *m*, and the second term is the average normalized recruitment of the remaining M−1 muscles, excluding muscle *m*.

Additionally, selectivity was analyzed at the level of specific target muscle groups, categorized into proximal and distal muscles of either the right or left leg. For this, the SI formula was adapted by averaging the normalized recruitment values across all target muscles in the group ( $REC_{T}$) and comparing them to the average recruitment of non-target muscles group. The selectivity index corresponding to the target muscle group is defined as

$$SI_{T}=REC_{T}-\frac{\sum_{n\notin M_{T}}^{M}REC_{n}}{M-M_{T}},$$ (6)

where $M_{T}$ denotes the set of target muscles in the group.

This index ranges from −1 to 1, enabling straightforward interpretation:

•
$SI_{T}=-1$: Target muscle group is minimally recruited compared to non-target muscles group.
•
$SI_{T}=0$: Target muscle group is equally recruited as non-target muscles group.
•
$SI_{T}=1$: Target muscle group is maximally recruited compared to non-target muscles group.

This analysis was conducted offline, following data acquisition through the automated stimulation protocol.

### D. Automated optimization of electrode position and stimulation amplitude for tSCS

After completion of the automated stimulation protocol across all electrodes, during which spinal reflexes were detected online within the predefined stimulation range and the corresponding peak-to-peak EMG responses were computed and stored, two independent automated algorithms were applied immediately thereafter. Using the near-instantaneously generated data, these algorithms determined the optimal electrode position ( $\varepsilon_{p}$) and stimulation amplitude ( $I_{\text{stim}}$) for each target muscle group. The first algorithm, the **ranking-based approach**, inspired by Salchow-Hömmen *et al.,*^27^ classifies responses based on a binary criterion (reflex present or absent). The second, the **selectivity-driven approach**, aims to maximize recruitment of the target muscle group by assessing the selective score $SI_{T}$.

These two approaches were selected based on established strategies in the tSCS literature: the RBA extends the binary reflex-based ranking introduced by Salchow-Hömmen *et al.*,^27^ while the SDA incorporates a quantitative selectivity metric following the formulation used by Bryson *et al.*,^26^ allowing explicit evaluation of targeted muscle-group recruitment.

(a) ***Ranking-Based Approach (RBA):***
  This method defines the most selective electrode position ( $\varepsilon_{p}$) and stimulation amplitude (*Istim*) for tSCS by applying a hierarchical set of prioritized rules based on the classification of reflex responses. For selectivity evaluation, the algorithm first determines the amplitude range in which fewer than half of the non-target muscle groups are activated. Within this selective amplitude range, the selection process is guided by the following criteria (1–4) for the target muscle groups:
  (1) Compare all electrodes and identify those in which at least 50% of the target muscles exhibit reflex responses (minimum requirement for consideration). Electrodes failing to meet this criterion are excluded from further selection.
  (2) Among the remaining electrodes, select the one that maximizes the number of target muscles exhibiting reflex responses. If multiple electrodes achieve the same number, proceed to the next criterion.
  (3) In the case of a tie, compare the stimulation amplitude at maximal response (*I*) and the amplitude at the first detected response ( $I^{\prime }$). Select the electrode that minimizes the difference between *I* and $I^{\prime }$, ensuring a more efficient response.
  (4) If a tie persists, select the electrode that operates at the lowest overall stimulation amplitude (*I*), which favors more energy efficient and comfortable stimulation.

The algorithm performs a ranking of all electrodes by applying these criteria sequentially, ultimately selecting the electrode (or electrodes, in the event of a tie) and the corresponding stimulation amplitude that best satisfies these conditions. The objective is to select the electrode that optimally stimulates the target muscle group while minimizing the activation of non-target muscles. In cases where no reflex responses are detected in the target muscle group within the defined selective amplitude range, the electrode is classified as non-selective.

(b) ***Selectivity-Driven Approach (SDA):***
  By leveraging recruitment curves, AUC metrics, and $SI_{T}$, the **SDA** identifies the optimal spinal electrode position ( $\varepsilon_{p}$) and stimulation amplitude (*Istim*) that maximize activation of target muscle groups while minimizing activation of non-target muscles. The SDA builds upon the foundation of the **RBA** by first selecting electrodes that meet a minimum criterion: at least 50% of the target muscles must be activated. If multiple electrodes satisfy this condition, the SDA compares their corresponding $SI_{T}$ values across all stimulation amplitudes.

To avoid selecting electrodes or amplitudes that produce negligible activation, the algorithm requires that at least 5% of the target muscle group is recruited at the evaluated amplitude. The $SI_{T}$ reflects the normalized recruitment of target muscles group relative to non-target muscles, offering a quantitative metric for selectivity. Electrodes with high $SI_{T}$ values and minimal overlap to non-target muscles are prioritized.

The logic underlying the SDA and RBA is illustrated in the flow chart shown in Fig. 3.

(c) ***Divergence Analysis of RBA and SDA using Normalized Relative Deviation:***
  To identify and quantify divergences in electrode selection outcomes between the two algorithms, we computed the normalized relative deviation (NRD) across both **midline and bilateral configurations**. First, we counted the number of cases where electrode selection differed. Then, we examined the **RBA** to identify potential weaknesses and sources of variability in its decision-making process. **Midline configuration** included 26 targets (13 participants × 2 muscle groups), while **bilateral configuration** covered 66 cases (11 participants × 6 muscle groups). The analysis followed these steps:
  (1) **Computing the Relative Difference:** For each condition ( $C_{1}$ to $C_{4}$), we calculated the difference between electrodes selected by both algorithms
$$\Delta_{i}=\frac{\left|E_{\text{RBA},i}-E_{\text{SDA},i}\right|}{E_{\text{SDA},i}},$$ (7)
    where $E_{\text{RBA},i}$ and $E_{\text{SDA},i}$ are the electrodes selected by each algorithm for condition *i*.
  (2) **Averaging Differences Per Condition:** The mean relative difference for each condition was computed as follows:
$${\bar{\Delta }}_{i}=\frac{1}{N_{i}}\sum_{j=1}^{N_{i}}\Delta_{ij},$$ (8)
    where $N_{i}$ is the total number of cases in condition *i*.
  (3) **Weighted Mean of Differences:** To assess the NRD, we computed a weighted average of ${\bar{\Delta }}_{i}$,
$$\text{NRD}=\frac{\sum_{i=1}^{4}\left(N_{i}\cdot {\bar{\Delta }}_{i}\right)}{\sum_{i=1}^{4}N_{i}}.$$ (9)
    This ensures that conditions with more occurrences have a greater influence on the final assessment.

The **NRD score** quantifies the variability in electrode selection. A **lower NRD** indicates greater consistency between algorithms, whereas a **higher NRD** represents greater divergence in electrode selection.

### E. Statistical analysis

Statistical analyses were performed separately for each target muscle group ( $SI_{T}$) to compare selectivity indices across spinal electrodes. In **midline and bilateral configuration**, each participant contributed one $SI_{T}$ per electrode per condition. Data normality was first assessed using the *Kolmogorov–Smirnov test*. As distributions were non-normal, we applied the *Kruskal–Wallis test* ( α=0.05) to detect overall differences in $SI_{T}$ across electrodes. Post hoc pairwise comparisons were conducted using the *Wilcoxon signed-rank test*, considering the within-subject structure. To correct for multiple comparisons, we applied the *Benjamini–Hochberg correction* ( α=0.05), ensuring statistical robustness.

## SUPPLEMENTARY MATERIAL

See the supplementary material for the additional experimental results and supplementary figures.

## Data Availability

The data that support the findings of this study are available from the corresponding author upon reasonable request and within the article and its supplementary material.
